# Supplementary material for: Exosome-mediated chemotaxis optimizes leader-follower cell migration
Source: PLoS Comput Biol. 2026 Jan 13;22(1):e1013894. doi: 10.1371/journal.pcbi.1013894 (PMC12826496; doi:10.1371/journal.pcbi.1013894)
Supplement: Attachment — Submitted filename: response.pdf [file pcbi.1013894.s001.pdf]

Response to referees for "Exosome-mediated chemotaxis optimizes leader-follower cell migration" (PCOMPBIOL-D-25-02085)

We thank the editor and the reviewers for their assessment, which has significantly improved our manuscript. Below, we provide point-by-point responses to the reviewers' comments.

Response to Reviewer #1

In this work, the authors study the migration of a follower cell in response to detecting chemotactic signals encapsulated in exosomes that are secreted from a leader cell. After setting up a stochastic models both for the movement of the follower cell and the secretion of exosomes, the authors show via explicit numerical simulations that there is an optimum in the average migration velocity of the follower cell as a function of the mean number of molecules in the exosomes. To rationalize this result, they consider an approximate model in 1D and show that this optimum behavior emerges naturally. This analysis also a reveals a non-dimensional parameter β that controls the migration of the follower cell.

The manuscript is well written, timely, and the results seem interesting.

I recommend publishing this study if the authors could consider (and answer) some of the points below.

1. The authors model both the follower cell and the exosomes as point particles. Yet the size a of the cell appears in their analysis. It is not clear where a appears in the modeling.

   In our simulations, a defines the physical capture radius of the cell. An exosome is detected, and its detection angle recorded, when it diffuses within this radius of the follower cell's position. This effectively treats the follower cell as having an absorbing boundary of size a for the signaling particles.

   We apologize that this was not clear from the manuscript. We have now created a Figure 1b that illustrates this rule (see below), and added the following text to the manuscript (line 95):

"The follower cell detects exosomes that reach within a capture radius a of the its position (x_f, y_f). This effectively treats the follower cell as having an absorbing boundary of size a."

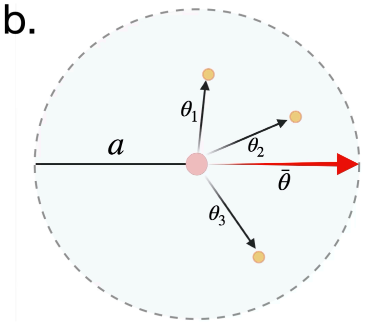

2. Both exosomes and the follower cells are assumed to move around freely by diffusion. The authors have neglected any elasticity or hindered diffusion in the extracellular matrix. Some justification (perhaps with numerical estimates) of why this is OK would be useful.

   The reviewer is correct that the extracellular matrix might reduce diffusion, especially for exosomes, whose diffusion constant is much faster than that of cells. Importantly, the range of exosome diffusion constants that we predict is best for chemotaxis is very broad (Figure 4b), extending even below the physiological range that we estimate for exosomes in aqueous environments based on their size ($D \sim 90-440$ μm^2/min). Therefore, we expect exosome-mediated chemotaxis to remain highly efficient even if the extracellular matrix reduces exosome diffusion.

   We have added this point to the text (line 258):

   "In fact, we see in Fig 4b that the follower cell velocity remains high even below this range, suggesting that chemotaxis would remain efficient even if exosome diffusion is reduced by interactions with the the extracellular matrix."

3. Related to the above point. Since the follower cell is a point particle, it is not clear what the authors mean by the arrival angle θ_i of the exosomes at the follower cell. This is important since the authors claim that the follower cell biases its motion towards the (fixed) leader cell. How is the directionality of the follower cell's movement determined? Do they have implicit global signaling gradients in mind?

   As illustrated in the new Figure 1b (see response 1 above), the arrival angle θ_i of each exosome is calculated based on its position with respect to the

cell center when it enters the capture radius a of the follower cell. We have added (line 115):

"The arrival angle is defined geometrically at the moment of detection as $\theta_i = \arctan((y_i - y_f)/(x_i - x_f))$*, where* (x_i, y_i) *is the exosome position and* (x_f, y_f) *is the follower cell position. This angle represents the direction on the cell membrane where the exosome signal is received."*

The directionality of the follower cell's movement is then determined by a weighted average of the θ_i, as illustrated in the new Figure 1b. The weighting is determined by the cargo size, arrival time, and arrival angle (Eq 7 in the text).

As the reviewer suggests, this mechanism functionally mimics the response to an internal polarity gradient informed by the history of localized detection events. We now point this out in the text (line 126) and cite a review on this topic [1].

4. I wonder if the average follower velocity in Fig2a ($H = 1$) is just an exponential function of the mean number of molecules in the exosome. This can be checked with a semilog plot. Similar considerations for the 'tail' of the plot in Fig2b ($H = 3$). And if so, can the authors provide a simple argument for the same?

A semi-log plot (below) indicates that these dependences may be exponential, although further investigation would be needed for a definitive conclusion. These curves (blue below) are for smaller follower velocities. In contrast, the dependence is linear for larger follower velocities (orange and green curves below—the linear dependence is more apparent on the linear scale, see Figs 2a and 2b; also see the red and yellow curves in 3a and 3b). Our model indeed predicts a linear dependence for large cargo size (Eq 17, 18). Therefore, we are satisfied with the model and leave further investigation to future work.

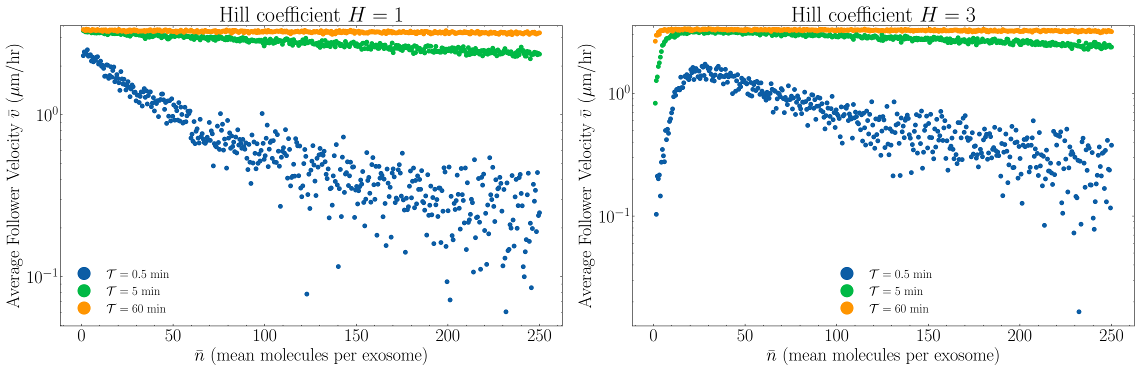

5. About the peak in the non-monotonic dependence of the average migration speed as a function of the mean number of molecules in the exosome (Fig2b). Isn't the peak just determined by the value of the saturation constant K in equation (4). As per table 1, $K = 25$ and the peak in Fig2b seems consistent with this observation. If this is the case, then isn't the switch-like behavior of the nonlinear activation function (equation 4) essentially the cause for this behavior?

   The reviewer is correct that the location of the peak is determined by the saturation constant K. We had stated this in the text (line 166) and confirmed it with our analysis (line 209). The reviewer is also correct as to the essential cause. We now add this reasoning to the text (line 168):

   "The optimum at larger H arises from the switch-like nature of the activation function (Eq 4): for $\bar{n} < K$, the cargo is insufficient to trigger the activation; whereas for $\bar{n} > K$, the response saturates, and further increasing the cargo size implies a penalty in exosome frequency. Thus, the optimum represents the most efficient packaging size that reliably exceeds the detection threshold."

6. Line 252, the authors claim that $H = 1$ is a linear detection regime. This is only true for $n \ll K$. Even with $H = 1$, the activation function is clearly nonlinear.

   Correct. We have changed 'linear' and 'nonlinear' there to 'cooperative' and 'non-cooperative,' respectively. We have also removed the terms 'linear' and 'nonlinear' elsewhere and simply defined the regimes by $H = 1$ and $H > 1$, respectively.

7. In the approximate 1D model, the directionality of movement is clear. However, this is not at all obvious in the simulations of the 2D. Related to point 3 above, it might help if the authors indicate (say in Fig 1) how direction sensing works in their study.

   As mentioned above, we have included the new Figure 1b (reproduced below). The caption reads:

   "(b) Direction sensing mechanism: The follower cell has a capture radius a. Exosomes detected within this radius are recorded as arrival vectors (thin arrows) defined by the angle θ_i relative to the cell center. The cell integrates these inputs to compute a weighted average direction $\bar{\theta}$ (thick red arrow) for migration."

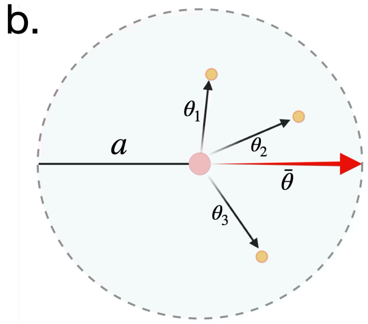

Response to Reviewer #2

In this manuscript, Gonzalez and Mugler analyze the plausibility of, and physical trade-offs inherent to, exosome-mediated directional signaling in cellular populations.

The authors have two primary results, both of which are well-supported by numerical and analytical arguments. The first is that cells face a trade-off between the number and "quality" of exosomes – the idea being that the cell has a finite number of signaling molecules to package into exosomes, and can either send out a few exosomes that are well-packed with signaling molecules, or many exosomes with fewer signaling molecules each. If the receiving cell requires a minimum number of signaling molecules per exosome to faithfully read the incoming information, then a clear optimum exists: the sending cell should send out as many exosomes as possible while keeping the mean number of signaling molecules/exosome at the minimum required to elicit a response in the receiving cell.

The second result is more subtle, and relates the diffusivity of the exosome to its capacity to carry meaningful directional information. The trade-off is well expressed by the authors: if an exosome diffuses too quickly, then it is of little value in indicating to the receiving cell which direction it came from. On the other hand, if it diffuses too slowly, it may be unable to find and collide with the receiver cell in the first place. The authors show, by exhaustive simulation, that this is indeed the case – an intermediate exosome diffusivity maximizes the speed of the receiving cell. Notably, the experimental range of exosome diffusivities coincides with the optimal range found in simulation.

These results are certainly interesting and new. Of the two, the latter is the most striking, though it is relatively underdeveloped compared to the first result. The first result is interesting, though arguably the trade-off is (a) straightforward, and (b) the result of modeling choices that are (as written) untethered to biological details.

My major questions are:

1. Could the authors provide some molecular detail to flesh out the plausibility of the first result? Are signaling molecules (e.g., chemoattractants) indeed packed within

exosomes (as opposed to packed on their surface), and what are the typical numbers (if known)?

Signaling molecules are found both within and on the surface of exosomes [2]. One way to distinguish between these two possibilities is to assay for the signaling molecule with and without digesting the exosome membrane. For example, a study employing this technique found the mRNA of CCL21 (an immune cell chemoattractant) inside exosomes, not on their surface [3]. Importantly, the "packet" assumption of our model holds regardless of whether the cargo is inside or surface-bound, as in both cases the molecules arrive collectively as a discrete unit. We have added this point to the text (line 27):

"Signaling molecules are found both within and on the surface of exosomes [2, 3]; in either case, signaling molecules arrive at the receiving cell in discrete packets."

As for typical numbers, for nucleic acids (e.g., miRNAs), the number can be quite low (often < 1 molecule per exosome on average [4]), whereas for proteins, exosomes can be densely packed. Given an exosome diameter of $30-150$ nm, a cargo load of $\bar{n} \approx 10-50$ molecules (the typical range in our model) corresponds to a concentration in the micromolar range, or a surface density of thousands of molecules per square micron, both of which are biophysically plausible. We have also added this point to the text (line 23):

"Experimental quantification reveals that cargo load can vary significantly: while some exosomes may carry sparse payloads (e.g., < 1 miRNA copy per vesicle on average [4]), others are enriched with high concentrations of signaling proteins such as chemokines [2]. This wide variability necessitates a theoretical approach that treats the mean cargo size as a tunable parameter."

2. Why couldn't the receiving cell simply use exosome fusion as a directional signal, rather than relying on a noisy read-out of chemoattractant number?

Using fusion itself would be less noisy, but the cargo provides specificity. Exosomes are a generic cell-cell communication platform, and the cargo distinguishes a chemotaxis signal from many other signals that an exosome could transmit. Indeed, experiments on exosome-mediated chemotaxis of cancer cells have shown that chemotaxis is not as efficient when exosomes are depleted of their cargo (fibronectin in that case) [5, 6]. We have added this clarification to the text (line 12):

"... cancer cells have been shown to migrate in response to exosomes, and to do so less efficiently when the exosomes are depleted of their chemical content [5, 6]."

3. The second part is compelling, but could benefit from some of the attention paid to the first part. For instance, could the authors determine what sets the optimal range of diffusivities?

We thank the reviewer for encouraging more attention to this part. We have now produced an analytic argument for the optimal diffusion coefficient and confirmed it with our simulations.

The analytic argument is as follows. The exosome signal must arrive at the follower cell by diffusion, even as the signal's source (the leader cell) moves away at speed v_0. Assuming that the two cells start with a separation l_0, this arrival time t satisfies $\sqrt{4Dt} = l_0 + v_0 t$, where $\sqrt{4Dt}$ is the typical distance covered by the diffusing exosomes, and $l_0 + v_0 t$ is the distance covered by the leader cell. We hypothesize that optimal diffusion sets this arrival time to maximize the signal difference across the follower cell's lengthscale a. There are two ways to obtain a timescale from a using the parameters above: $t \sim a/v_0$ or $t \sim a^2/D$. Inserting either $t = a/v_0$ or $t = a^2/D$ into our arrival condition and solving for the diffusivity obtains, respectively, $D^* = v_0(l_0 + a)^2/(4a)$ or $D^* = v_0 a^2/(2a - l_0)$. The latter is negative since $l_0 > 2a$, and therefore only the former makes physical sense.

Whereas a and v_0 are biophysical parameters that are experimentally well characterized, the initial cell separation l_0 can vary. Our expression $D^* = v_0(l_0 + a)^2/(4a)$ predicts that the optimal exosome diffusivity increases with initial cell separation, which makes sense because otherwise the signal would not reach the follower cell before the leader cell got too far away.

To test this prediction, we ran many simulations like those in Fig 4b for various initial cell separations l_0. At each value of l_0, we determined the diffusion coefficient that maximizes the follower velocity. The results are shown by the black data below (mean and standard error over 5 trials). The prediction is plotted in red, and we see good agreement, especially since the prediction is a heuristic argument that only aims to capture the basic physics.

This plot has been added as Figure 4c, and the above discussion has been added to the text (line 267).

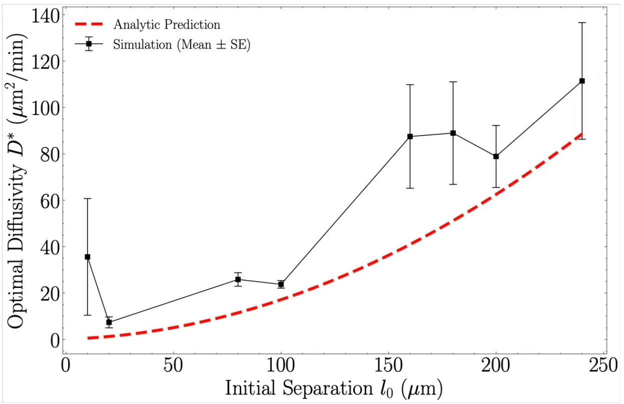

4. While it may not make sense to study cell migration in five spatial dimensions, how do these results change with the geometry of the space that cells move in? (e.g., in a channel of variable width – I presume that for small enough widths, there is not downside to rapid diffusion?)

This is a good question, as confinement is relevant in physiological environments. We actually suspected that the downside to rapid diffusion would persist in a channel with small width: this would confine the cells to a quasi-one-dimensional space, but infinite diffusion would still erase the signal because the follower cell would have equal probability to go left or right.

To test this hypothesis, we added confinement of variable width to our simulations. Two reflective walls (for both follower cell and exosomes) were placed parallel to the leader cell's movement direction at variable distances L on either side of the leader cell. As shown below, we saw very little change in the follower dynamics, including the persistence of an optimal exosome diffusivity, even as the wall distance was reduced to ten cell radii a.

This plot has been added as Figure 4d, and the above discussion has been added to the text (line 288).

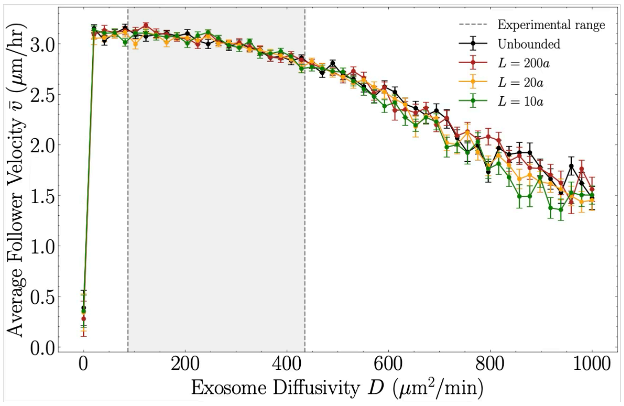

5. Finally, in the Discussion, the authors state:

   "First, they demonstrate that exosome-packaged signals can rival the information content of smooth molecular gradients, provided that cargo size, secretion rate, and memory integration are properly tuned."

   This is quite a strong claim, and as far as I can tell is not justified by the results in this manuscript. Could the authors provide some more context and/or comparison of the two modes of chemotaxis?

   We agree with the reviewer and have removed that sentence.

Response to Reviewer #3

In this manuscript, the authors present a computational model for exosome-mediated chemotaxis. Analysis of the model reveals that it produces a non-monotonic dependence of the migration velocity on the exosome cargo size. The modeling approach and analysis seem technically sound.

1. However, my concern with the paper is that I don't see any evidence in the references cited by the authors for cells detecting chemical gradients through the uptake of diffusing exosomes.

   We had cited references from two experimental groups that provide evidence for cells detecting chemoattractants via exosomes, and now we have moved this content to the first paragraph of the introduction to make the experimental motivation completely clear.

   Specifically, it has been demonstrated that Dictyostelium amoeba cells secrete exosomes containing the chemoattractant cAMP that guide the migration of other cells [7]. Furthermore, it has been demonstrated that cancer cell chemotaxis is actively driven by exosome secretion, and that efficient chemotaxis depends on the exosomes' chemical cargo [5, 6].

   Thus, we have added the following text to the first paragraph of the Introduction (line 10):

   "For instance, amoeba cells have been shown to secrete exosomes containing the chemoattractant cAMP that guide the migration of other cells [7]. Similarly, cancer cells have been shown to migrate in response to exosomes, and to do so less efficiently when the exosomes are depleted of their chemical content [5, 6]."

2. And it is not clear how this would work. Chemical gradients are detected by receptors located on the cell surface. If the contents of exosomes are taken up by the cell, how is the cell able to use this information to detect a gradient?

   Whether the exosome binds to surface receptors or fuses, the interaction occurs at a specific location on the cell membrane (defined in our model by the arrival angle θ_i). If the mechanism is receptor-mediated, the signaling cascade initiates at the point of contact. If the mechanism involves fusion and uptake, the cargo is released locally at the fusion site. This creates a transient concentration of signaling molecules in the cytoplasm near the membrane before they diffuse globally throughout the cell. The problem of maintaining spatial information with internal diffusing readouts is also faced in the usual context of gradient detection by receptors, for example in the "local excitation, global inhibition" (LEGI) mechanism of chemotaxis [8].

   We have added this clarification to the text (line 97):

   "Whether the exosome binds to surface receptors or fuses, the interaction occurs at a specific location on the follower cell's membrane. Therefore, we assume that the cell has a mechanism to retain and integrate information about the arrival location of the exosome, similar to how cells retain and integrate that information for single-molecule chemoreception."

3. One potentially interesting role for exosomes in gradient sensing has been suggested by Arya et al. (Nat. Cell Bio., 2025, 27:931). In their model, DNA secreted from neutrophils ensnares LTB4-containing exosomes. This creates a spatial gradient of LTB4 that can be detected by migrating neutrophils and macrophages. I think this would be an interesting scenario to model to investigate if it provides advantages over the direct release of LTB4.

   We thank the reviewer for bringing this very interesting study to our attention. We now describe it in the Discussion (line 331) as a potential future direction for our work:

   "Further studies of exosome-mediated chemotaxis in heterogeneous environments may be warranted. For example, recent work demonstrates that neutrophils can secrete DNA networks that physically ensnare LTB4-containing exosomes, thereby creating a stable, high-fidelity gradient for follower cells [9]. This mechanism effectively immobilizes the signal packets ($D \to 0$) after secretion, preventing diffusive dissipation. Extending our model to account for such spatially variable diffusivity or trapping mechanisms could reveal further design principles by which cells structure their communication landscape."

References

[1] Ghose D, Elston T, Lew D. Orientation of cell polarity by chemical gradients. Annual review of biophysics. 2022;51(1):431–451.

[2] Xue D, Zheng Y, Wen J, Han J, Tuo H, Liu Y, et al. Role of chemokines in hepatocellular carcinoma. Oncology Reports. 2021;45(3):809–823.

[3] Feng Y, Zhong X, Ni HF, Wang C, Tang TT, Wang LT, et al. Urinary small extracellular vesicles derived CCL21 mRNA as biomarker linked with pathogenesis for diabetic nephropathy. Journal of Translational Medicine. 2021;19(1):355.

[4] Chevillet JR, Kang Q, Ruf IK, Briggs HA, Vojtech LN, Hughes SM, et al. Quantitative and stoichiometric analysis of the microRNA content of exosomes. Proceedings of the National Academy of Sciences. 2014;111(41):14888–14893.

[5] Sung BH, Ketova T, Hoshino D, Zijlstra A, Weaver AM. Directional cell movement through tissues is controlled by exosome secretion. Nature communications. 2015;6(1):7164.

[6] Sung BH, Weaver AM. Exosome secretion promotes chemotaxis of cancer cells. Cell Adhesion & Migration. 2017;11(2):187–195.

[7] Kriebel PW, Majumdar R, Jenkins LM, Senoo H, Wang W, Ammu S, et al. Extracellular vesicles direct migration by synthesizing and releasing chemotactic signals. Journal of Cell Biology. 2018;217(8):2891–2910.

[8] Xiong Y, Huang CH, Iglesias PA, Devreotes PN. Cells navigate with a local-excitation, global-inhibition-biased excitable network. Proceedings of the National Academy of Sciences. 2010;107(40):17079–17086.

[9] Arya SB, Collie SP, Xu Y, Fernandez M, Sexton JZ, Mosalaganti S, et al. Neutrophils secrete exosome-associated DNA to resolve sterile acute inflammation. Nature Cell Biology. 2025; p. 1–17.
